# Triterpenoids Extracted From *Antrodia cinnamomea* Mycelia Attenuate Acute Alcohol-Induced Liver Injury in C57BL/6 Mice *via* Suppression Inflammatory Response

**DOI:** 10.3389/fmicb.2020.01113

**Published:** 2020-07-03

**Authors:** Yange Liu, Zhuqian Wang, Fange Kong, Lesheng Teng, Xiaoyi Zheng, Xingkai Liu, Di Wang

**Affiliations:** ^1^School of Life Sciences, Jilin University, Changchun, China; ^2^School of Basic Medical Sciences, Nanchang University, Nanchang, China; ^3^Division of Nephrology, Stanford University School of Medicine, Stanford, CA, United States; ^4^Hepatobiliary and Pancreatic Surgery, The First Hospital of Jilin University, Jilin University, Changchun, China

**Keywords:** *Antrodia cinnamomea* mycelia, triterpenoids, alcohol, liver injury, inflammatory response

## Abstract

Excessive alcohol consumption causes liver injury–induced mortality. Here we systematically analyzed the structure of triterpenoids extracted from *Antrodia cinnamomea* mycelia (ACT) and investigated their protective effects against acute alcohol-induced liver injury in mice. Liquid chromatography–mass spectrometry and liquid chromatography with tandem mass spectrometry were performed to determine the structures of ACT constituents. Alcohol-induced liver injury was generated in C57BL/6 mice by oral gavage of 13 g/kg white spirit (a wine at 56% ABV). Mice were treated with either silibinin or ACT for 2 weeks. Liver injury markers and pathological signaling were then quantified with enzyme-linked immunosorbent assays, antibody array assays, and Western blots, and pathological examinations were performed using hematoxylin-eosin staining and periodic acid–Schiff staining. Triterpenoids extracted from *A. cinnamomea* mycelia contain 25 types of triterpenoid compounds. A 2-weeks alcohol consumption treatment caused significant weight loss, liver dyslipidemia, and elevation of alanine aminotransferase, aspartate aminotransferase, γ-glutamyl transferase, and alkaline phosphatase activities in the serum and/or liver. These effects were markedly reversed after 2-weeks ACT administration. Triterpenoids extracted from *A. cinnamomea* mycelia alleviated the organ structural changes and inflammatory infiltration of alcohol-damaged tissues. Triterpenoids extracted from *A. cinnamomea* mycelia inhibited proinflammatory cytokine levels and enhanced anti-inflammatory cytokine levels. Acute alcohol treatment promoted inflammation with significant correlations to hypoxia-inducible factor 1α (HIF-1α), which was reduced by ACT and was partially related to modulation of the protein kinase B (Akt)/70-kDa ribosomal protein S6 kinase phosphorylation (p70S6K) and Wnt/β-catenin signaling pathways. In conclusion, ACT protected against acute alcohol-induced liver damage in mice mainly through its suppression of the inflammatory response, which may be related to HIF-1α signaling.

## Introduction

According to a World Health Organization report on alcohol and health in 2018, alcohol abuse kills more than three million people each year. Excessive alcohol consumption is the most frequent cause of alcoholic liver disease (ALD), which involves alcoholic hepatitis, steatosis, steatohepatitis, fibrosis, and cirrhosis (Gonçalves et al., 2017). Acute alcoholic hepatitis and liver cirrhosis are associated with a high mortality rate, which can reach 50% in acute alcohol hepatitis. Although low-grade fatty liver disease can be alleviated after alcohol withdrawal, 35% of heavy alcohol drinkers will develop more severe forms of liver injury (Lucey et al., 2009). Alcoholic liver disease imposes a significant and increasing treatment burden on society.

Excessive levels of alcohol and alcohol metabolites upregulate the levels of cytokine/chemokine receptors and proinflammatory cytokines including tumor necrosis factor (TNF), interferons (IFNs), and interleukins (ILs) (Gao and Bataller, 2011; Wang et al., 2018). The spleen, an important source of proinflammatory cytokines, is consistently damaged in patients with ALD (Cesta, 2006). Alcohol metabolism causes central venous hypoxia, which results from increased oxygen consumption and decreased oxygen delivery to the liver (Tsukamoto and Xi, 2010). Under hypoxic conditions, hypoxia-inducible factor 1α (HIF-1α) facilitates the synthesis of nitric oxide (NO), increases the expression of cytokines such as TNF-α, and promotes inflammation and cell death (Pan et al., 2018). All of these processes are involved in ALD and especially in alcoholic hepatitis. Depletion of HIF-1α in hepatocytes can alleviate alcohol-induced fat accumulation and inflammation in the liver (Nath et al., 2011). This evidence indicates that there is an association between inflammation and HIF-1α and that HIF-1α may be a potential therapeutic target for ALD treatment.

Medicines commonly used to treat acute alcoholic hepatitis, such as metadoxine, s-ademetionine, and silibinin, exert various side effects that limit their efficacies (Ambade et al., 2018). Certain fungi and their natural products can potentially function as novel medicines because of their pharmacological effectiveness and reduced side effects. We have previously demonstrated that *Antrodia cinnamomea*, an edible fungus used in traditional medicine, exerts hepatoprotective effects by modulating oxidative stress (Liu et al., 2017b). More than 78 compounds, including terpenoids, have been extracted from *A. cinnamomea* mycelium through submerged fermentation, and the potential pharmaceutical activities of some of these compounds have been evaluated (Ma et al., 2014). Triterpenoids are derived from squalene or related acyclic 30-carbon precursors, are the largest and most structurally diverse group of natural products, and are regarded as the most important biologically active natural products besides polysaccharides (Yu et al., 2010). The hepatoprotective qualities of *A. cinnamomea* and its triterpenoid compounds against CCl_4_- and N-nitrosodiethylamine–induced liver injury in mice have been studied (Tien et al., 2017). Although the hepatoprotective qualities of *A. cinnamomea* against alcohol-induced liver injury have been reported, only antrosterol (Chang et al., 2017) and antroquinonol (Kumar et al., 2011) have been extracted from *A. cinnamomea* and its fruiting body (Lu et al., 2007; Huang et al., 2010). The secondary metabolites of petri dish–cultured *A. cinnamomea* can reduce aspartate aminotransferase (AST)– and alanine aminotransferase (ALT)–related pathologies and hepatic fat accumulation upon alcohol-induced liver injury (Wu et al., 2019). However, an association between the triterpenes contained in cultured mycelia of *A. cinnamomea* and hepatoprotection has not yet been established.

In this study, we aimed to systemically determine the structure of triterpenoids extracted from *A. cinnamomea* mycelia grown by submerged fermentation and to investigate the hepatoprotective properties and underlying mechanisms of action in mice with acute alcohol injury.

## Materials and Methods

### *A. cinnamomea* Culture and Sample Preparation

*Antrodia cinnamomea* mycelia were obtained by submerged fermentation as previously described (Liu et al., 2017a). Triterpenoids were extracted in 80% ethanol twice at 80°C for 100 min. After centrifugation, the supernatant was collected and concentrated at 50°C. After being diluted ninefold in D.D. water, the samples were loaded onto an AB-8 type Amberlyst column (3 × 45 cm) and eluted with D.D. water and 40, 80, and 100% ethanol, respectively, at a flow rate of 0.8 mL/min. The eluent fractions from 80 and 100% ethanol elutions were collected. The triterpenoids of *A. cinnamomea* mycelia (ACT) were prepared by removing the ethanol from wash solution. Vanillin-glacial acetic acid and perchloric acid colorimetric spectrophotometry was performed to analyze the concentrations of the triterpenoids as previously described (Ma et al., 2014).

### Liquid Chromatography–Mass Spectrometry and Liquid Chromatography With Tandem Mass Spectrometry Analysis

**Figure 1:**
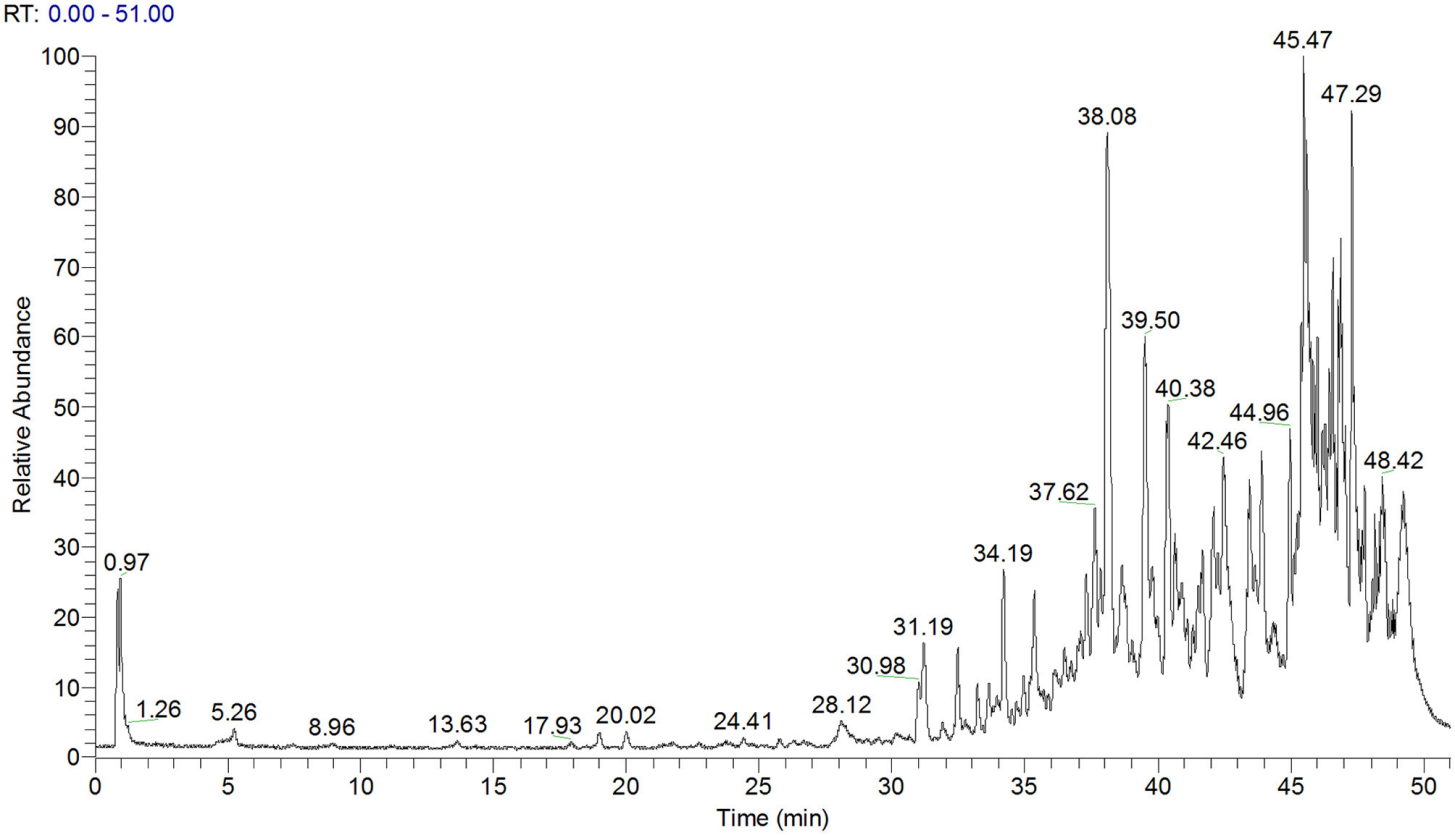
Total ion chromatogram of ACT as obtained by LC-MS. ACT, triterpenoids separated from *A. cinnamomea* mycelia.

Approximately 50 mg of ACT was extracted with 800 μL of methanol and 10 μL of internal standard (2.9 mg/mL, dl-*o*-chlorophenylalanine). The solution was ground using a grinding mill at 65 Hz for 45 s, vortexed for 30 s, and then centrifuged at 12,000 revolutions/min for 15 min at 4°C. Subsequently, 200 μL of supernatant was transferred to a vial for liquid chromatography–mass spectrometry (LC-MS) analysis (UltiMate 3000LC, Orbitrap Elite; Thermo Fisher Scientific, Waltham, MA, USA) with a Hypersil GOLD C18 column (100 × 2.1 mm, 1.9 μm; Thermo Fisher Scientific, Waltham MA, USA). Chromatographic separation conditions were as follows: column temperature: 40°C; flow rate: 0.3 mL/mL; mobile phase A: water + 0.1% formic acid; mobile phase B: acetonitrile + 0.1% formic acid; injection volume: 4 μL; automatic injector temperature: 4°C. ESI–: ESI-heater temperature: 300°C; sheath gas flow rate: 45 arb; aux gas flow rate: 15 arb; sweep gas flow rate; 1 arb; spray voltage: 3.2 kV; capillary temperature: 350°C; S-Lens RF level: 60%; liquid chromatography with tandem mass spectrometry (LC-MS/MS): high-energy collision dissociation, 40 V; resolution: 17,500. The gradient of the mobile phase is shown in Table 1S. The details of mass spectra detected by LC-MS/MS from the ACT sample are shown in Figure 1S.

### Animal Experiment Design

**Figure 2:**
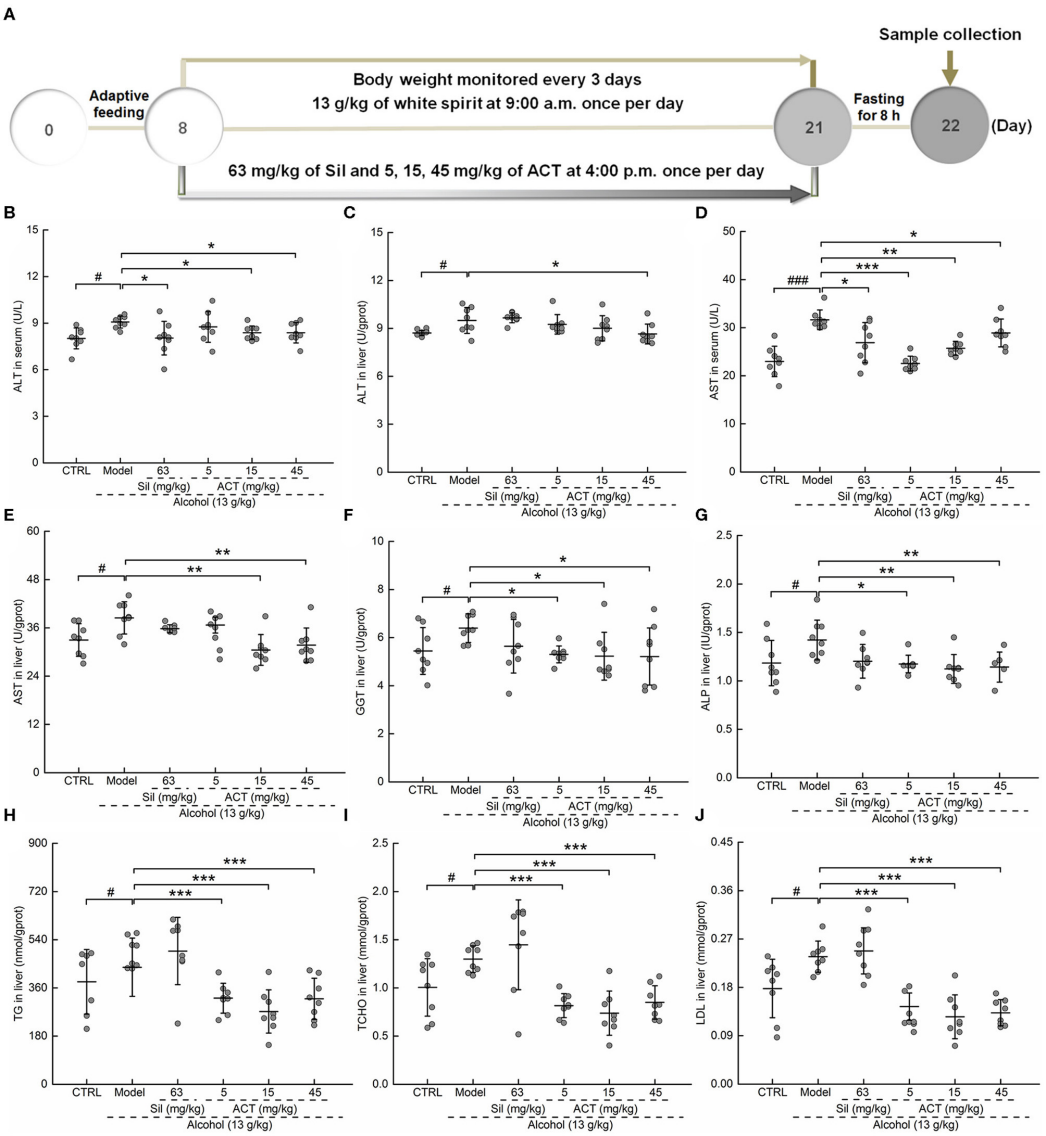
ACT treatment decreases liver injury markers and lipid abnormalities. Mice were treated with ACT for 14 days, and their liver injury markers were quantified with enzyme-linked immunosorbent assay. **(A)** The experimental protocol and drug administration. **(B–F)** The levels of **(B,C)** alanine aminotransferase and **(D,E)** aspartate aminotransferase in both the serum and liver, respectively, and the levels of **(F)** γ-glutamyl transferase, **(G)** alkaline phosphatase, **(H)** triglyceride, **(I)** total cholesterol, and **(J)** low-density lipoprotein in the liver. Data are expressed as the means ± *SD* (*n* = 8) and were analyzed using a one-way analysis of variance with parametric tests. ^#^*P* < 0.05 and ^###^*P* < 0.001 vs. the control group; *P < 0.05, **P < 0.01, and ***P < 0.001 vs. the model group. ACT, triterpenoids separated from *A. cinnamomea* mycelia; Sil, silibinin.

The study protocol was approved by the Institutional Animal Ethics Committee of Jilin University (no. SY0605). The model development of alcohol-induced liver injury was similar to that described in previous studies with some modification (Huang et al., 2015; Lim et al., 2015; Massey et al., 2018). Eight-weeks-old C57BL/6 male mice (18–22 g) were purchased from Liaoning Changsheng Biotechnology Co., Ltd. [SCXK (Liao)-2015-0001, Liaoning, China] and housed in a controlled room with a relative humidity of 50 ± 5%, a temperature of 23 ± 1°C, and a 12-h light/dark cycle. After 7-days acclimatization, all mice were randomized into six groups (*n* = 8/group). One group of mice (control group, *n* = 8) was treated (oral gavage) with 10 mL/kg normal saline (0.9% NaCl), and the other five groups of mice were treated (oral gavage) with 13 g/kg of white spirit (a wine at 56% ABV; Beijing Shunxin Agricultural Co. Ltd., Beijing, China) at 9:00 am once per day. At 4:00 P.M, on the same day, the control group (*n* = 8) and one experimental group (*n* = 8) were treated (oral gavage) with 10 mL/kg normal saline; one group (*n* = 8) was additionally treated (oral gavage) with 63 mg/kg of silibinin (Sil group) (Tianjin Tasly Sants Pharmaceutical Co. Ltd., Tianjin, China), used as a positive control; and three groups were additionally orally gavaged with 5 mg/kg (*n* = 8), 15 mg/kg (*n* = 8), or 45 mg/kg (*n* = 8) of ACT. The treatment period lasted for 14 days, and all mice were weighed every 3 days. A detailed experimental protocol and drug administration specifics are shown in Figure 2A.

### Sample Collection

After the last administration, the mice were fasted for 8 h. Blood was collected from the retrobulbar plexus/sinus. Tissues including those of the liver, kidney, spleen, and heart were quickly harvested after euthanasia.

### Histology Assay

The collected tissues were fixed in 10% buffered formalin overnight, dehydrated in an ethanol series, cleared with dimethyl benzene, embedded in paraffin, and cut into 5-μm-thick sections. To assess pathological changes, sections of all organs were stained with hematoxylin-eosin (H&E), and the sections of the kidneys were further stained with periodic acid–Schiff (PAS) stain. All stained slides were examined using an IX73 inverted microscope (400×; Olympus, Tokyo, Japan).

### Antibody Array Assay

A total of 111 cytokines, chemokines, and growth factors were detected in the liver of mice with acute alcohol injury using the Proteome Profiler Mouse XL Cytokine Array kit (ARY028; R&D Systems, Minneapolis, MN, USA) according to the manufacturer's protocols. Briefly, 200 μg of each sample was diluted with the array buffer. The membranes were blocked with 2 mL of array buffer for 1 h and then incubated with the prepared samples overnight at 4°C with rocking. After washing, the membranes were incubated with primary antibody for 1 h, followed by incubation with streptavidin/horseradish peroxidase for 30 min at room temperature. The membranes were then exposed using the developing substrate solution under the chemiluminescence imager (Chemi Scope 6300; Clinx Science Instruments, Shanghai, China).

### Enzyme-Linked Immunosorbent Assay

Based on cytokine screening from the antibody array, the levels of the following cytokines and enzymes in the serum, liver, and/or spleen were measured using an enzyme-linked immunosorbent assay (ELISA) kit (Shanghai Yuanye Bio-Technology Co. Ltd., Shanghai, China) according to the manufacturer's instructions: human cartilage glycoprotein 39 (YKL-40, CK-E95772), plasminogen activator inhibitor 1 (PAI-1, CK-E93562), ALT (CK-E90314), AST (CK-E90386), chemokine (C-X-C motif) ligand 13 (CXCL13, CK-E95658), thrombopoietin (TPO, CK-E93965), retinol-binding protein 4 (RBP4, CK-E20170), γ-glutamyl transferase (GGT, CK-E94933), alkaline phosphatase (ALP, CK-E20105), IL-7 (CK-E20125), IL-22 (CK-E93411), IL-33 (CK-E94161), IL-1α (CK-E20009), vascular endothelial growth factor (VEGF, CK-E20260), regulated upon activation normal T cell expressed and secreted (RANTES, CK-E20198), P-selectin (CK-E20212), intercellular cell adhesion molecule 1 (ICAM-1, CK-E11381), neutrophil gelatinase-associated lipocalin (NGAL, CK-E20174), vascular cell adhesion molecule 1 (VCAM-1, CK-E20253), NO (CK-E20293), reactive oxygen species (ROS, CK-E91516), TNF-α (CK-E20220), IFN-α (CK-E20311), IFN-β (CK-E20334), triglyceride (TG, CK-E91733), total cholesterol (TCHO, CK-E91839), and low-density lipoprotein (LDL, CK-E91911).

### Western Blot Analysis

Liver and spleen tissues were homogenized in cold radioimmunoprecipitation assay (Sigma-Aldrich, St. Louis, MO, USA) buffer containing 1% protease inhibitor cocktail (Sigma-Aldrich) and 2% phenylmethanesulfonyl fluoride (Sigma-Aldrich). The protein concentrations of lysed tissues were analyzed using a bicinchoninic acid protein assay kit (Merck Millipore, Burlington, MA, USA). Subsequently, 40–50 μg of protein was separated using 10–12% sodium dodecyl sulfate–polyacrylamide gel electrophoresis and transferred to polyvinylidene difluoride membranes. After blocking with 5% bovine serum albumin, the membranes were incubated at 4°C overnight with the following antibodies: phosphorylated (P)-protein kinase B (Akt, ab131443), total (T)-Akt (ab200195), P-mammalian target of rapamycin (mTOR, ab109268), T-mTOR (ab32028), P-glycogen synthase kinase 3β (GSK-3β, ab75745), T-GSK-3β (ab93926), T-70-kDa ribosomal protein S6 kinase (p70S6K, ab184551), P-β-catenin (ab27798), T-β-catenin (ab32572), Wnt 1 (ab85060), Wnt 3+3α (ab172612), HIF-1α (ab16066) (Abcam, Cambridgeshire, UK), P-p70S6K (#9234; CST, Boston, USA), and glyceraldehyde-3-phosphate dehydrogenase (GAPDH, ABS16; Merck Millipore). After washing, the membranes were incubated with horseradish peroxidase–conjugated goat anti–rabbit secondary antibodies (bs-0295G; Beijing Biosynthesis Biotechnology Co. Ltd., Beijing, China). Proteins were visualized using a Gel Imaging System (UVP, Upland, CA, USA), and the band intensities were quantified using Image J (National Institutes of Health, Bethesda, MD, USA).

### Statistical Analysis

Data are expressed as the means ± *SD* and were analyzed using SPSS 16.0 software (IBM, Armonk, NY, USA). Comparisons between groups were performed using a one-way analysis of variance with parametric test, following by *post-hoc* multiple comparisons (Dunn test). *P* < 0.05 was considered as statistically significant.

Liquid chromatography–MS data analysis for feature extraction was performed and preprocessed with Compound Discoverer software (Thermo Fisher Scientific) and then normalized and edited.

## Results

### Major Triterpenoid Types in ACT

As determined by LC-MS data (Figure 1) and according to the United Network of Human Metabolome Database, 25 major types of triterpenoids were found in ACT (Table 1). Their detailed mass spectra from LC-MS/MS are shown in Figure 1S.

**Table 1:** Twenty-five major types of triterpenoids extracted from ACT and analyzed by LC-MS and LC-MS/MS.

**No**.	**Molecular weight**	**Name**	**CAS**	**MZ**	**RT (min)**	**Area: ACT.raw (F1)**
1	436.3339	Ganoderal A	104700-98-3	435.3266	45.63	1535.710215
2	438.3496	Ganoderol A	104700-97-2	437.3423	45.631	44145.19097
3	440.3278	Camelledionol	81426-90-6	439.3205	47.147	1041.589219
4	440.3655	Ganoderol B	104700-96-1	439.3582	45.374	538.4306751
5	442.3439	Camellenodiol	81426-91-7	441.3366	48.075	4116.106675
6	448.3180	Porrigenin A	196607-75-7	447.3107	46.447	982.1673132
7	452.3290	Tyromycic acid	104759-35-5	451.3217	45.169	3005.389475
8	454.3437	Ganoderal B	114020-55-2	453.3364	48.011	811.0190589
9	456.3599	Ganodermanondiol	/	455.3526	48.549	8811.756089
10	460.2807	Lucidenic acid N	364622-33-3	459.2735	45.964	298.8221905
11	462.2977	Lucidenic acid M	110241-33-3	461.2904	35.382	1294.49912
12	468.3242	Glabrolide	10401-33-9	467.3169	45.541	1176.825686
13	469.2101	**Desoxylimonin**	/	468.2028	45.448	645.6217662
14	470.3392	Rubinic acid	94662-96-1	469.3319	45.187	20425.83661
15	470.3755	Momoridcin	/	469.3683	47.359	43707.50927
16	484.3182	Ganolucidic acid E	114567-50-9	483.3109	38.929	2069.389669
17	488.3495	Ganoderiol D	114567-45-2	487.3422	38.393	29266.99928
18	490.3653	Ganoderiol H	114612-72-5	489.3580	46.104	1090.990007
19	502.3293	Ganolucidic acid B	98683-75-1	501.3221	46.002	4160.083098
20	502.3652	**Ganoderiol I**	114567-49-6	501.3579	40.078	24539.03582
21	504.3446	Protobassic acid	37905-13-8	503.3373	47.996	19431.66385
22	504.3813	**Ganoderiol G**	114567-48-5	503.3741	37.971	1841.985826
23	516.3445	Phytolaccinic acid	54928-05-1	515.3372	46.269	733.9864822
24	528.3808	Tsugaric acid B	201045-20-7	527.3735	46.134	2656.340618
25	544.1966	**Physalin D**	54980-22-2	543.1893	38.42	1721.83703

*Bold text indicates no found fragment ion. ACT, triterpenoids separated from A. cinnamomea mycelia; CAS, chemical abstracts service; MZ, mass-to-charge ratio; RT, retention time*.

### Protection by ACT Against Alcohol-Induced Liver Injury

ACT treatment in mice reversed the body weight loss induced by excessive alcohol, especially on the fourth, and 7th day (*P* < 0.05; Table 2S).

Alcohol increased the activity of ALT (*P* < 0.05, Figures 2B,C) and AST (Figures 2D,E) in both the serum and liver and increased the levels of GGT (*P* < 0.05; Figure 2F) and ALP (*P* < 0.05; Figure 2G) in the liver, indicating early liver injuries. Alcohol treatment in mice increases the levels of TG (*P* < 0.05; Figure 2H), TCHO (*P* < 0.05; Figure 2I), and LDL (*P* < 0.05; Figure 2J), suggesting alcohol-driven dyslipidemia. Only ACT, but not Sil, attenuated these upregulated lipid metabolism products in mice with acute alcohol injuries (*P* < 0.001; Figures 2H–J). Triterpenoids extracted from *A. cinnamomea* mycelia administered at 15 and 45 mg/kg showed similar efficacy on pathological changes after a 14-days administration (*P* < 0.05; Figures 2B–J). However, ACT at 15 mg/kg failed to influence the hepatic levels of ALT (Figure 2C).

**Figure 3:**
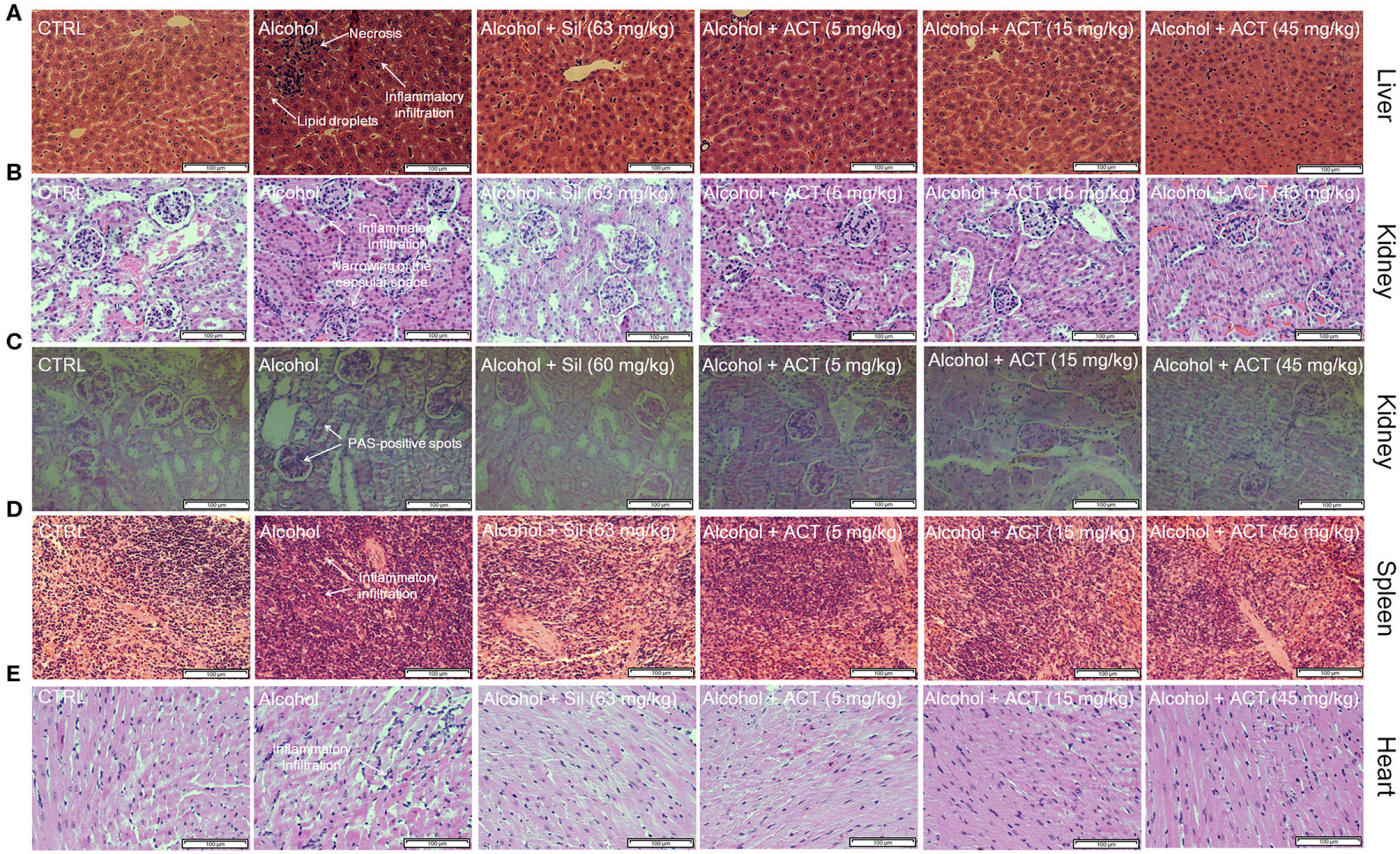
ACT treatment alleviates structural organ changes and hepatocyte apoptosis. Histopathological analyses of the **(A)** liver, **(B)** kidney, **(D)** spleen, and **(E)** heart by H&E staining, and of the **(C)** kidney by PAS staining (scale bar 100 μm; magnification × 400). ACT, triterpenoids separated from *A. cinnamomea* mycelia; Sil, silibinin; H&E, hematoxylin and eosin; PAS, periodic acid–Schiff.

Compared with the control mice, the alcohol-injured mice exhibited characteristic damage to the liver including lipid droplets, inflammatory infiltration, and necrosis (Figure 3A), symptoms that were relieved by ACT administration (Figure 3A). Triterpenoids extracted from *A. cinnamomea* mycelia treatment also alleviated alcohol-induced injuries such as thickening of the basement membrane, narrowing of the capsular space (Figure 3B), a high rate of PAS-positive spots (Figure 3C) in the kidney, and an increased ratio of inflammatory infiltration in the spleen (Figure 3D) and heart (Figure 3E). Compared with the healthy control mice, 45 mg/kg of ACT administration showed no effect on the pathological features of the liver, spleen, kidney, or heart, suggesting that the use of ACT is safe for these organs (Figure 2S).

### Effects of ACT on the Inflammatory Response in Acute Alcohol-Injured Mice

ACT administered at 15 and 45 mg/kg showed similar efficacies on the pathological changes in cytokines and organ structures. According to the recommended dosage of *A. cinnamomea* for human consumption and the extraction ratio of triterpenoids in this study, we chose liver samples obtained from mice treated with 15 mg/kg ACT for analysis by cytokine screening.

**Figure 4:**
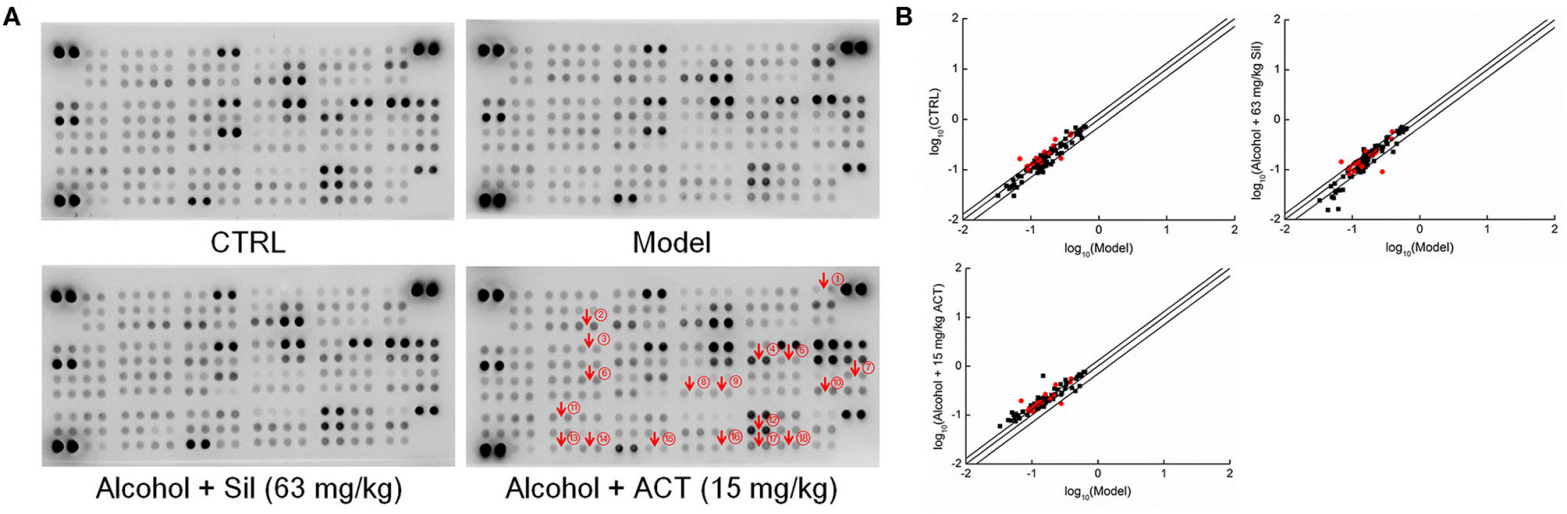
Antibody array assay. The effects of ACT and Sil on 111 cytokines in the livers of mice with acute alcohol-induced liver injury were detected using the Proteome Profiler Mouse XL Cytokine Array kit (*n* = 3). **(A)** The array graph represents the cytokine expressions. **(B)** Scatter diagram of the 111 cytokines. The relative density is the ratio of the absolute value and the reference spot value. The red dots indicate the proteins validated by enzyme-linked immunosorbent assay. ACT, triterpenoids separated from *A. cinnamomea* mycelia; Sil, silibinin. ① RANTES, regulated upon activation normal T cell expressed and secreted; ② YKL-40, human cartilage glycoprotein 39; ③ CXCL13, chemokine (C-X-C motif) ligand 13; ④ ICAM-1, intercellular cell adhesion molecule 1; ⑤ IFN-γ, interferon γ; ⑥ IL-1α, interleukin 1α; ⑦ IL-7, interleukin 7; ⑧ IL-22, interleukin 22; ⑨ IL-23, interleukin 23; ⑩ IL-33, interleukin 33; ⑪ NGAL, neutrophil gelatinase-associated lipocalin; ⑫ RBP4, retinol-binding protein 4; ⑬ P-selectin; ⑭ PAI-1, plasminogen activator inhibitor 1; ⑮ TPO, thrombopoietin; ⑯ TNF-α, tumor necrosis factor α; ⑰ VCAM-1, vascular cell adhesion molecule 1; ⑱ VEGF, vascular endothelial growth factor.

Using the data obtained from the antibody array assay, 21 of the 111 target cytokines were chosen for further analysis based on their correlation with ALD and their changes among experimental groups (Figure 4 and Table 3S). Compared with the acute alcohol-injured mice, Sil-treated mice showed increased levels of 14 cytokines and reduced levels of seven cytokines, whereas ACT-treated mice (15 mg/kg) showed increased levels of 20 cytokines and reduced level of one cytokine in the liver tissues (Figure 4 and Table 3S).

The ELISA method was used to validate the changes in cytokines identified by the cytokine array. Compared with the acute alcohol-injured mice, ACT-treated mice showed reduced levels of 12 cytokines including IL-1α (*P* < 0.05), IL-7 (*P* < 0.05), IL-33 (*P* < 0.05), TNF-α (*P* < 0.05), IFN-α (*P* < 0.01), IFN-β (*P* < 0.05), VEGF (*P* < 0.05), RANTES (*P* < 0.01), P-selectin (*P* < 0.05), CXCL13 (*P* < 0.05), YKL-40 (*P* < 0.05), and PAI-1 (*P* < 0.05) and increased levels of IL-22 (*P* < 0.05) in the liver (Table 2).

**Table 2:** ACT and Sil regulates the expression level of inflammatory cytokines in liver of mice with acute alcohol exposure.

	**CTRL**	**Alcohol**	**Alcohol + Sil (mg/kg)**	**Alcohol** **+** **ACT (mg/kg)**		
			**63**	**5**	**15**	**45**
NO (μmol/gprot)	7.7 ± 2.5	10 ± 2.3^#^	9 ± 4.9	6.6 ± 1.5^*^	4.1 ± 1.1^**^	5.6 ± 1.1^**^
ROS (U/mgprot)	353.2 ± 35.7	392.8 ± 38.2^#^	353.9 ± 18.2^*^	371.8 ± 29.7	336.9 ± 32^**^	342.9 ± 21^**^
IL-1α (pg/mgprot)	5.7 ± 1.5	6.3 ± 0.8	5.4 ± 1.6	4.5 ± 0.2^**^	4.8 ± 0.4^**^	5 ± 1^*^
IL-7 (pg/mgprot)	30 ± 2.2	32.6 ± 1.8^#^	30.6 ± 2.6	30.9 ± 2.2	28.9 ± 1.6^*^	30.5 ± 2.8
IL-22(pg/mgprot)	5.3 ± 0.8	4.6 ± 0.4^#^	5.5 ± 1.7	4.6 ± 0.1	5.1 ± 0.8	5.3 ± 0.7^*^
IL-33 (pg/mgprot)	15.5 ± 2.9	22.9 ± 4.4^##^	16.4 ± 4.1^**^	15.9 ± 1.6^**^	17.3 ± 2.6^*^	18.6 ± 4.8^*^
TNF-α (pg/mgprot)	69.4 ± 12.7	81.7 ± 6.8^#^	74.7 ± 17.5	75.8 ± 7.2	67.1 ± 15.2^*^	68.6 ± 12.4^*^
IFN-α (pg/mgprot)	6.1 ± 1.3	7.2 ± 0.7^#^	5.7 ± 1.2^*^	5.7 ± 0.4^**^	5.4 ± 0.9^**^	5.4 ± 0.7^***^
IFN-β (pg/mgprot)	69.6 ± 6.9	88.8 ± 14.9^##^	80.2 ± 23.7	75.2 ± 7.8	79 ± 8.2	72 ± 7.9^*^
VEGF (pg/mgprot)	29.6 ± 5.3	39 ± 10.1^#^	32.6 ± 7.5	32.7 ± 4.3	33.2 ± 4.1	30.7 ± 3.9^*^
RANTES (pg/mgprot)	70.1 ± 13.2	83.9 ± 6.5^#^	68.5 ± 7.9^**^	67 ± 5.4^**^	70.1 ± 15.6	60.1 ± 7.2^**^
P-selectin (pg/mgprot)	22 ± 5.4	31 ± 5.2^##^	25.2 ± 7.4	31.4 ± 1.6	25.3 ± 2^*^	27.8 ± 2.4
CXCL13 (pg/mgprot)	142.9 ± 15.5	158.5 ± 16.4^#^	147.2 ± 23.2	145.5 ± 10.9	149.5 ± 12.5	138.7 ± 14.1^*^
YKL-40 (ng/mgprot)	14.5 ± 0.8	15.2 ± 0.6^#^	14.4 ± 0.8^*^	14.9 ± 0.3	14.3 ± 1.1^*^	14.2 ± 0.6^**^
PAI-1 (pg/mgprot)	175.3 ± 24.9	194.7 ± 14.1^#^	184 ± 18.3	179.5 ± 13.1	182.2 ± 13.1	177.5 ± 18.7^*^

*Data are expressed as the means ± SD (n = 8) and were analyzed using a one-way analysis of variance (ANOVA) with parametric tests*.

# P <0.05 and

## *P <0.01 vs. the control group (one-way ANOVA)*;

* *P <0.05*,

** P <0.01, and

*** *P <0.001 vs. the model group (one-way ANOVA). ACT, triterpenoids separated from A. cinnamomea mycelia; Sil, silibinin*.

Furthermore, a 14-days treatment of ACT at 45 mg/kg resulted in 22.5, 19.3, 28.5, 23.7, 40.9, and 36.9% reductions in the levels of IL-1α (*P* < 0.05), IL-7 (*P* < 0.05), IL-33 (*P* < 0.05), TNF-α (*P* < 0.05), IFN-α (*P* < 0.01), and IFN-β (*P* < 0.05), respectively, compared with their levels in acute alcohol-injured mice, with the exception of IL-22 in the spleen (Table 3).

**Table 3:** ACT and Sil regulates the expression level of inflammatory cytokines in the spleen of mice with acute alcohol-exposure.

	**CTRL**	**Alcohol**	**Alcohol + Sil (mg/kg)**	**Alcohol** **+** **ACT (mg/kg)**		
			**63**	**5**	**15**	**45**
NO (μmol/gprot)	5.9 ± 1.5	9.4 ± 1.6^##^	7.1 ± 1.8^*^	7.8 ± 0.9	7.9 ± 1.2	6.1 ± 1.7^**^
ROS (U/mgprot)	170.9 ± 23.7	218.7 ± 79.2^#^	240.9 ± 51.4	201.3 ± 31	155 ± 36^*^	122.3 ± 13.4^**^
IL-1α (pg/mgprot)	12.3 ± 3.5	16 ± 3.2^#^	14 ± 3.4	16.1 ± 1.2	15 ± 3	12.4 ± 3.3^*^
IL-7 (pg/mgprot)	34.6 ± 7	49.2 ± 7.8^###^	40.3 ± 9.2^*^	43.4 ± 8.2	49 ± 8.2	39.7 ± 8.3^*^
IL-22 (pg/mgprot)	13.2 ± 3.3	10.2 ± 0.9	8.8 ± 2.4	10 ± 2.4	9.9 ± 2.6	10.1 ± 2.5
IL-33 (pg/mgprot)	35.6 ± 9	52.7 ± 12^##^	41.3 ± 12	40.7 ± 7.2	43 ± 6	37.7 ± 9.2^*^
TNF-α (pg/mgprot)	11.6 ± 1.9	15.6 ± 3.7^##^	11.7 ± 3.3	11.9 ± 1.5^*^	14.4 ± 2	11.9 ± 1.7^*^
IFN-α (pg/mgprot)	12.2 ± 1.5	16.4 ± 5.8^#^	12.5 ± 3.9	12.9 ± 2.2	11 ± 2.8	9.7 ± 2.7^**^
IFN-β (pg/mgprot)	163.7 ± 15.5	286.2 ± 93.9^##^	217.7 ± 28	184.9 ± 17.7	201.9 ± 41.4	180.6 ± 26.5^*^

*Data are expressed as the means ± SD (n = 8) and were analyzed using a one-way analysis of variance (ANOVA) with parametric tests*.

# *P <0.05*,

## P <0.01 and

### *P <0.001 vs. the control group (one-way ANOVA)*;

* P <0.05 and

** *P <0.01 vs. the model group. ACT, triterpenoids separated from A. cinnamomea mycelia; Sil, silibinin*.

NO and ROS are major molecules involved in the pathogenesis of inflammatory diseases (Liu et al., 2018b), and the hepatic and splenic levels of NO and ROS were significantly reduced by ACT treatment at 45 mg/kg in acute alcohol-injured mice (*P* < 0.01; Tables 2, 3).

ACT and Sil failed to affect the hepatic levels of TPO, RBP4, IL-23, ICAM-1, NGAL, and VCAM-1 in acute alcohol-injured mice compared with the model group (Table 4S).

### Effects of ACT on the Expression of HIF-1α, Akt, and Wnt1/β-Catenin Signaling-Related Proteins

**Figure 5:**
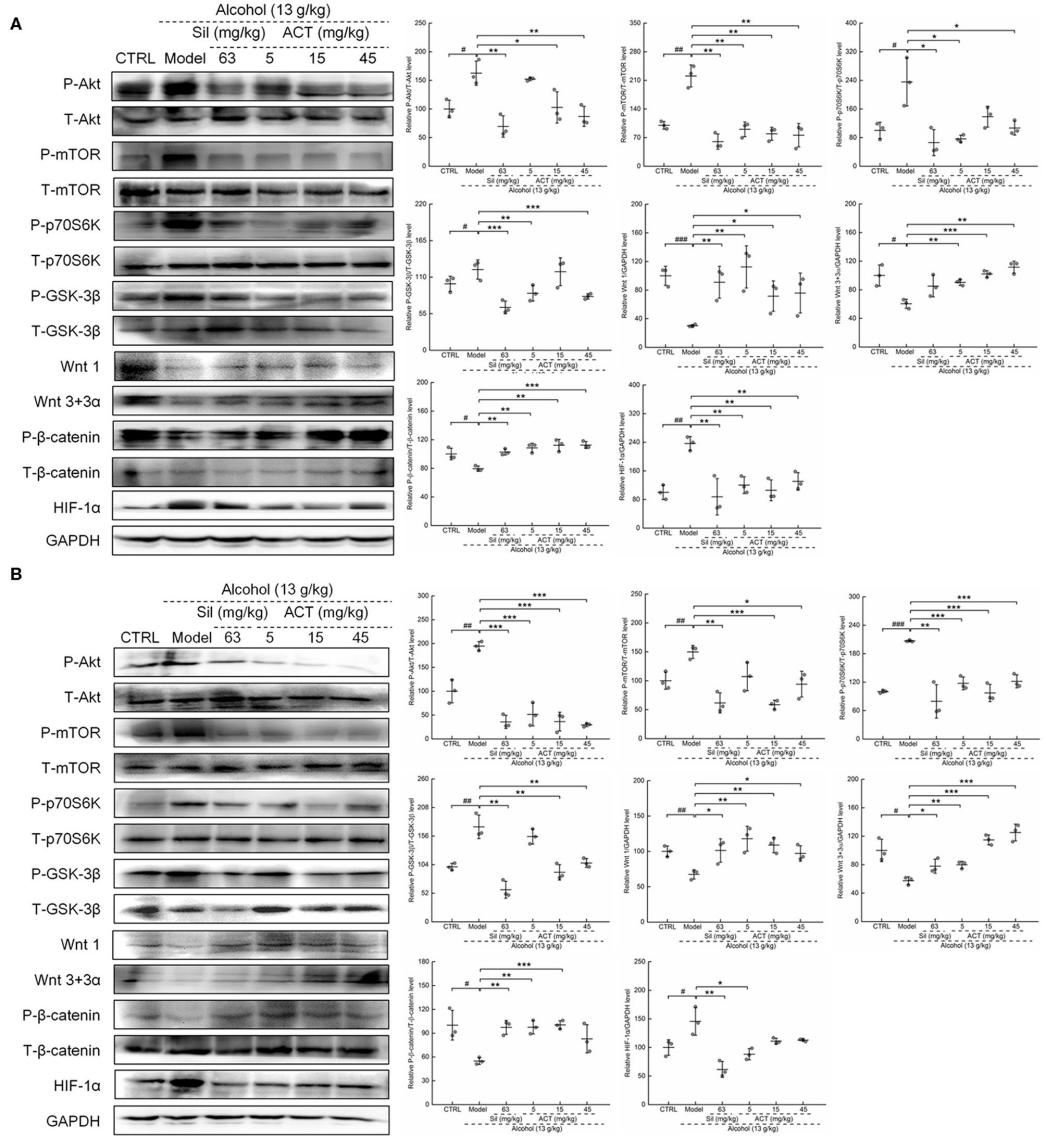
ACT treatment regulates HIF-1α *via* Akt/p70S6K and Wnt/β-catenin signaling in the liver and spleen. Fourteen-day ACT administration regulated the phosphorylation levels of Akt, mTOR, GSK-3β, p70S6K, and β-catenin and the expression levels of Wnt 1, Wnt3, and HIF-1α in the liver **(A)** and spleen **(B)** of mice with acute alcohol-induced injuries. Quantified protein expression was normalized by related total protein expression and/or GAPDH expression. Data are expressed as the means ± *SD* (*n* = 3) and were analyzed using a one-way analysis of variance with parametric tests. ^#^*P* < 0.05 and ^##^*P* < 0.01 vs. the control group; *P < 0.05, **P < 0.01, and ***P < 0.001 vs. the model group. ACT, Triterpenoids separated from *A. cinnamomea* mycelia; Sil, silibinin; Akt, protein kinase B; mTOR, mammalian target of rapamycin; GSK-3β, glycogen synthase kinase 3 beta; p70S6K, 70-kDa ribosomal protein S6 kinase; GAPDH, glyceraldehyde-3-phosphate dehydrogenase.

Based on the data obtained from the antibody array assay, we hypothesized that the HIF-1α expression may be involved in ACT-mediated hepatoprotection against acute alcohol-induced liver injury. We then analyzed the protein expression levels of HIF-1α and its important upstream regulators Akt/p70S6K and Wnt/β-catenin. Alcohol consumption significantly increased the protein levels of HIF-1α (*P* < 0.05) and the phosphorylation levels of Akt (*P* < 0.05), mTOR (*P* < 0.05), p70S6K (*P* < 0.05), and GSK-3β (*P* < 0.01) (Figures 5A,B). Triterpenoids extracted from *A. cinnamomea* mycelia treatment also reduced the protein levels of Wnt1 (*P* < 0.05) and Wnt 3+3α (*P* < 0.01) and the phosphorylation levels of β-catenin (*P* < 0.01) in the liver (Figure 5A) and spleen (Figure 5B) of mice with acute alcohol injury. These pathological alterations in the protein levels and phosphorylation statuses were remarkably alleviated over 14 days of ACT administration, especially at doses of 15 and 45 mg/kg. Comparatively, Sil failed to regulate the expression levels of Wnt 3+3α in the liver (Figure 5A) of mice with acute alcohol injuries.

## Discussion

The triterpenoids are a large and structural diverse group of important natural compounds that are abundant in *A. cinnamomea* and have well-established biological activities (Yu et al., 2010). We elucidated the structures of these chemical constituents by LC-MS and LC-MS/MS and confirmed the presence of 25 types of triterpenoid compounds in ACT. Some types of triterpenoids have been confirmed to show protective effects on liver cells. Ganodermanondiol has shown beneficial effects on t-BHP–induced hepatotoxicity through an AMPK- and Nrf2-mediated pathway in human liver-derived HepG2 cells (Li et al., 2013). Ganoderol B has also shown inhibitory effects in HepG2 cells against H_2_O_2_-induced increases in ALT and AST levels (Peng et al., 2013), and physalin D has been shown to effectively clear 1,1-diphenyl-2-picrylhydrazyl radicals and exert antioxidant activity (Helvaci et al., 2010). Our data provide a complete list of ACT triterpenoids, which may be potential medicines to treat liver diseases in the future.

Alcohol consumption results in changes in the cell membrane permeability, leading to the leakage of hepatic ALT and AST into the blood. The occurrence of liver damage is often accompanied by abnormal increases in ALT, AST, GGT, and ALP activities (Zhang et al., 2017), which serve as the clinical indicators to determine the liver function status (Nada et al., 2010). Lipid metabolism disorders in the liver, such as pathological changes in the levels of TG, TCHO, and LDL, can also be used for the diagnosis of ALD (Zheng et al., 2014). In our study, ACT treatment suppressed the high levels of ALT and AST, remedied the defects of liver lipid metabolism, and alleviated the pathological changes in liver tissues, thus confirming its protective activity against acute alcohol-induced liver injury. Our data suggest that ACT is a candidate medicine to restore hepatocyte membrane permeability and lipid metabolism in cases of alcohol-induced liver injury.

Furthermore, ACT relieved the alcohol-induced inflammatory cell infiltration of organs in our ALD mice, suggesting that it plays an important role against inflammatory responses. According to the antibody array assay, 15 mg/kg ACT treatment strongly influenced the levels of 21 cytokines, most of which are associated with inflammatory responses. Indeed, studies have also reported that the alcohol metabolites acetaldehyde and acetate can directly induce an inflammatory response (Wang et al., 2012) *via* TNF-α overexpression and cause mitochondrial electron transport chain dysfunction, leading to the production of ROS as a by-product (Shanmugam et al., 2003; Shen et al., 2009). The generation of ROS induces the secretion of YKL-40 (Gerin et al., 2016), which is highly expressed in patients with liver fibrosis and cirrhosis (Shen et al., 2009). Reactive oxygen species and TNF-α can promote the release of PAI-1 (Zagotta et al., 2013) and promote cell necrosis *via* the activation of inflammatory responses (Wang et al., 2012), which further helps in the release of IL-1α (Iyer et al., 2009), a factor responsible for neutrophil infiltration (Eigenbrod et al., 2008; Hajime et al., 2010). P-selectin, the expression of which is also controlled by TNF and IL-1, predominantly mediates venular leukocyte recruitment to the sites of inflammation in the liver, similar to the progression of acute allergic responses (Klintman et al., 2004; Sun et al., 2012). The production of NO can be stimulated by IL-33, which is highly expressed in patients with ALD and serves as an alarm during severe tissue injury (Wang et al., 2017), and is responsible for the formation of hydroxyl radicals (Liu et al., 2017b) that impair mitochondrial respiration during alcohol-induced liver damage (Hao et al., 2018). Patients with ALD also show high levels of RANTES and CXCL13. RANTES is involved in the inflammatory processes during the progression of liver fibrosis (Karatayli et al., 2018), and CXCL13 controls circulation and redistribution of B cells in lymphoid organs (Murray et al., 2017). In our study, the anti-inflammatory effects of ACT were confirmed by the suppression of these proinflammatory cytokines, selectins, and ROS after ACT treatment in acute alcohol-injured mice, and these effects may play important roles in hepatoprotection against alcohol-induced acute liver injury.

Alcohol consumption induces hypoxia in the centrilobular area of the liver (French, 2004), which has been considered to promote cellular inflammatory responses, toxicity, and apoptosis (Yun et al., 2014). Interferon α and TNF-α control the expression level of hypoxia-stabilized HIF-1α (Tsapournioti et al., 2013; Yeh et al., 2018), which further regulates the levels of ROS (Yu et al., 2018) and PAI-1 (Zagotta et al., 2013). In addition, under hypoxic conditions, ROS produced from complex III of the respiratory chain are mediated by HIF-1α *via* the reduction of prolyl hydroxylase activity (Chua et al., 2010). Notably, HIF-1α binds to the hypoxia-responsive element in the promoters of target genes to mediate the inflammatory response (Wang et al., 2013) and promote alcohol-induced lipid accumulation (Nath et al., 2011), which can worsen liver injury. Our results showed that the HIF-1α expression was markedly elevated after alcohol administration, similarly to the previous findings (Yun et al., 2014), and was inhibited by 14-days ACT treatment.

Hypoxia activates mTOR signaling, which is triggered by phosphorylation *via* the Akt pathway. Upregulation of mTOR and its downstream target gene p70S6K, which in turn activates HIF-1α, exacerbates ischemia/reperfusion-induced liver inflammation (Zhu et al., 2018). Increased phosphorylation of Akt can suppress the activation of GSK-3β by upregulating its phosphorylation (Huang et al., 2015), and the inhibited activation of GSK-3β is responsible for the accumulation of HIF-1α (Cheng et al., 2014). Activated GSK-3β can regulate β-catenin ubiquitination and degradation, which in turn can be disabled by Wnt ligand binding. Alcohol consumption induces hepatocyte damage by promoting the upregulation of Foxo3A, which suppresses Wnt/β-catenin signaling (Huang et al., 2015), and the activation of Wnt/β-catenin promotes hepatocyte survival under hypoxic conditions (Liu et al., 2018a). Our results suggest that ACT mediates these changes in the expression of proteins involved in the Akt/p70S6K and Wnt/β-catenin pathways, which together regulate the HIF-1α expression. Additionally, hypoxia is considered to promote cellular inflammatory responses. In this study, ACT exerted protective effects against acute alcohol-induced liver injury mainly by preventing inflammatory responses, which may be related to the reduction of the overexpression of HIF-1α signaling.

There are certain limitations to this investigation. First, ACT treatment at 15 and 45 mg/kg showed similar efficacy in regulating the levels of cytokines and proteins in the livers of alcohol-treated mice. Triterpenoids extracted from *A. cinnamomea* mycelia treatment at 45 mg/kg showed greater effects on the levels of cytokines in the spleen than that at 15 mg/kg. Two-weeks administration of ACT at 45 mg/kg also showed few adverse effects on healthy mice, indicating its safety for use in treating liver injury. More experiments are warranted to determine the optimal dosage of ACT treatment for hepatoprotection against alcohol damage. Second, ACT contains 25 types of triterpenoids, and we did not determine which specific ACT triterpenoids caused the hepatoprotective effects observed in this study. Preliminary results from *in vitro* analysis suggest that the hepatoprotective effects may be caused by several types of triterpenoids working together. Third, HIF-1α serves as a junction between alcohol-induced hypoxia and the inflammatory response. However, we analyzed the expression changes in proteins related only to HIF-1α signaling. Further experiments are warranted in HIF-1α siRNA-transfected cells and knockout mice to further confirm the relationship between Wnt/β-catenin, Akt/p70S6K, and HIF-1α.

By systematic determination of the triterpenoids contained in ACT, we verified the protective properties of ACT against alcohol-induced liver injury in mice, which may be related to the ACT-induced modulation of the HIF-1α expression. These data establish that the hepatoprotective effects of ACT occur mainly through the suppression of the inflammatory response, which may be related to HIF-1α signaling.

## Data Availability Statement

All datasets generated for this study are included in the article/Supplementary Material.

## Ethics Statement

The animal study was reviewed and approved by the Institution Animal Ethics Committee of Jilin University (NO. SY0605).

## Author Contributions

DW and XL conceived and design of research. DW and XZ edited and revised manuscript. XL approved final version of manuscript. YL performed experiments and drafted manuscript. ZW analyzed data and prepared figures. FK and LT interpreted results of experiments. All authors contributed to the article and approved the submitted version.

## Conflict of Interest

The authors declare that the research was conducted in the absence of any commercial or financial relationships that could be construed as a potential conflict of interest.
