# Supplementary material for: Triterpenoids Extracted From Antrodia cinnamomea Mycelia Attenuate Acute Alcohol-Induced Liver Injury in C57BL/6 Mice via Suppression Inflammatory Response
Source: Front Microbiol. 2020 Jul 3;11:1113. doi: 10.3389/fmicb.2020.01113 (PMC7350611; doi:10.3389/fmicb.2020.01113)
Supplement: Supplementary file 1 [file Data_Sheet_1.pdf]

Supplementary Material

MATERIAL AND METHODS

The study protocol was approved by the Institution Animal Ethics Committee of Jilin University (NO. SY0605). Eight-week-old C57BL/6 male mice (18-22 g) are purchased from Liaoning Changsheng Biotechnology Co., Ltd (SCXK (Liao)-2015-0001, Liaoning, China) and housed in a controlled room with a relative humidity of 50±5%, temperature of 23±1℃ and a cycle of 12 h light/dark. After a 7-day acclimatization, the mice were randomly divided into two groups, and orally gavaged with 10 mL/kg silane serving as control mice (CTRL) (n=6) and 45 mg/kg of ACT (n=6) for 14 days. After euthanasia, the organs including liver, spleen, kidney and heart were collected.

H&E staining was used to analyze the pathological changes of the collected organs.

Table list

Table 1s. The gradient of mobile phase.

Time (min)	Flow rate (mL min^{-1})	A (%)	B (%)	Time (min)	Flow rate (mL min^{-1})	A (%)	B (%)
0	0.3	98	2	43	0.3	55	45
10	0.3	90	10	44	0.3	10	90
25	0.3	80	20	47	0.3	10	90
30	0.3	70	30	48	0.3	98	2
40	0.3	55	45	51	0.3	98	2

Table 2s. Effects of ACT treatment on the bodyweight of mice with acute alcohol-induced liver injury.

	CTRL	Alcohol	Alcohol + Sil (mg/kg)	Alcohol + ACT (mg/kg)		
			63	5	15	45
1st	21.9±1	22±1	21.7±1.6	21.9±1.1	21.7±1.1	21.6±1.2
4th	22.4±0.9	18.1±1.2###	18.5±1.4	18.6±1.9	19.2±1.6*	18.9±1.1*
7th	21.5±0.7	17.8±1.5###	18.9±1.2	18.5±2.7	19.6±1.5**	19.4±1.4**
10th	22.4±1.1	18.9±1.6###	19.3±1.4	19.3±1.5	18.8±1.6	19.4±1.5
13rd	22.8±1	19.1±1.3###	19.2±1.8	19.8±1.2	20.2±1.2*	19.6±1.3

Data are expressed as the means ±S.D. (n=8) and were analyzed using a one-way analysis of variance (ANOVA) with parametric tests. ### *P* < 0.001 *vs.* the control group; * *P* < 0.05 and ** *P* < 0.01 *vs.* the model group. ACT: Triterpenoids separated from *A. cinnamomea* mycelia; Sil, Silibinin.

Table 3s. The detail parameters of target cytokines upregulated or downregulated among experimental groups.

Coordinate	Target	Fold (vs. Model)			Coordinate	Target	Fold (vs. Model)		
		CTRL	63 mg/kg Sil	15 mg/kg ACT			CTRL	63 mg/kg Sil	15 mg/kg ACT
A1-A2	Reference Spots	0	0	0	F5-F6	IGFBP-6	-2.805	2.785	26.300
A3-A4	Adiponectin/Acrp30	-35.640	7.592	12.864	**F7-F8**	**IL-1 alpha/IL-1F1**	**12.176**	**1.514**	**16.210**
A5-A6	Amphiregulin	-32.007	-34.086	16.997	F9-F10	IL-1 beta/ IL-1F2	2.664	-7.384	19.740
A7-A8	Angiopoietin-1	-30.725	-27.719	15.338	F11-F12	IL-1ra/IL-1F3	50.012	-27.939	-32.255
A9-A10	Angiopoietin-2	-24.474	-25.188	26.000	F13-F14	IL-2	8.947	-64.462	79.785
A11-A12	Angiopoietin-like 3	10.495	6.682	25.748	(F15-F16	IL-3	1.343	-46.131	59.771
A13-A14	BAFF/BLyS/TNFSF13B	-46.138	-39.098	65.178	F17-F18	IL-4	2.877	-23.449	20.642
A15-A16	C1q R1/CD93	-28.572	-73.881	59.045	F19-F20	IL-5	-7.491	-25.431	18.576
A17-A18	CCL2/JE/MCP-1	-16.536	-49.102	89.790	F21-F22	IL-6	-9.882	-43.048	125.697
A19-A20	CCL3/CCL4 MIP-1 alpha/beta	1.497	-25.935	62.615	**F23-F24**	**IL-7**	**11.274**	**-13.554**	**33.839**
**A21-A22**	**CCL5/RANTES**	**-40.060**	**-67.564**	**-38.562**	G1-G2	IL-10	5.798	2.660	42.534
A23-A24	Reference Spots	0	0	0	G3-G4	IL-11	5.538	2.900	33.609
B3-B4	CCL6/C10	-28.475	-27.087	18.140	G5-G6	IL-12p40	7.504	-3.114	40.861
B5-B6	CCL11/Eotaxin	-26.586	-28.447	8.593	G7-G8	IL-13	6.583	3.655	29.496
B7-B8	CCL12/MCP-5	-31.405	-28.100	9.309	G9-G10	IL-15	13.610	0.899	18.586
B9-B10	CCL17/TARC	-11.795	-17.743	19.364	G11-G12	(IL-17A)	16.088	-40.852	32.369
B11-B12	CCL19/MIP-3 beta	-17.303	-10.634	22.680	**G13-G14**	**(IL-22)**	**29.531**	**-10.020**	**45.114**
B13-B14	CCL20/MIP-3 alpha	-23.787	-1.879	60.307	**G15-G16**	**(IL-23)**	**23.628**	**-13.573**	**38.277**
B15-B16	CCL21/6Ckine	36.001	35.515	58.024	G17-G18	IL-27p28	-4.763	-24.922	24.771
B17-B18	CCL22/MDC	12.744	-13.249	33.597	G19-G20	IL-28	-6.010	-17.168	10.731
B19-B20	CD14	2.874	-20.661	57.470	**G21-G22**	**IL-33**	**27.819**	**18.916**	**83.752**
B21-B22	CD40/TNFRSF5	-15.281	-44.070	-11.824	G23-G24	LDL R	29.357	19.943	19.465

C3-C4	(CD160)	-25.416	-24.017	19.147	H1-H2	Leptin	5.591	2.033	44.593
C5-C6	(Chemerin)	-30.783	-29.294	1.246	H3-H4	LIF	9.021	-3.257	29.035
**C7-C8**	**Chitinase 3-like 1/YKL-40**	**39.652**	**45.421**	65.546	**H5-H6**	**Lipocalin-2/NGAL**	**138.606**	**107.196**	**182.965**
C9-C10	Coagulation Factor III/ Tissue Factor	-15.765	22.538	34.145	H7-H8	(LIX)	37.938	15.112	18.625
C11-C12	Complement Component C5/C5a	-9.565	7.243	29.000	H9-H10	(M-CSF)	1.727	7.828	10.979
C13-C14	Complement Factor D	-25.186	0.408	0.269	H11-H12	(MMP-2)	0.780	23.840	7.577
C15-C16	C-Reactive Protein/CRP	13.003	26.008	29.991	H13-H14	(MMP-3)	-12.544	-37.239	31.612
C17-C18	CX3CL1/Fractalkine	0.230	-13.394	56.126	H15-H16	(MMP-9)	-7.083	-26.885	79.309
C19-C20	CXCL1/KC	-11.716	-34.558	85.842	H17-H18	Myeloperoxidase	70.096	32.704	37.903
C21-C22	CXCL2/MIP-2	-27.395	-37.277	11.759	H19-H20	Osteopontin (OPN)	2.487	18.077	39.695
D1-D2	CXCL9/MIG	17.780	-15.456	-5.363	H21-H22	Osteoprotegerin/ TNFRSF11B	-14.007	-4.886	58.931
D3-D4	CXCL10/IP-10	-1.889	0.682	29.266	H23-H24	PD-ECGF/ Thymidine phosphorylase	-24.265	0.258	11.335
D5-D6	CXCL11/I-TAC	-3.215	-2.499	26.007	I1-I2	PDGF-BB	-2.413	12.492	8.957
**D7-D8**	**CXCL13/BLC/BCA-1**	**41.973**	**7.748**	**35.558**	I3-I4	Pentraxin 2/SAP	22.160	19.273	49.333
D9-D10	CXCL16	0.417	5.925	18.684	I5-I6	Pentraxin 3/ TSG-14	32.285	21.941	50.078
D11-D12	Cystatin C	7.700	26.858	18.200	I7-I8	Periostin/OSF-2	13.675	-5.112	58.056
D13-D14	DKK-1	11.883	2.369	49.846	I9-I10	Pref-1/DLK-1/FA1	21.467	35.303	-2.584
D15-D16	DPPIV/CD26	18.446	3.954	24.376	I11-I12	Proliferin	9.559	12.400	7.626
D17-D18	EGF	-13.512	-21.879	24.745	I13-I14	Proprotein Convertase 9/ PCSK9	13.578	25.058	20.569
D19-D20	Endoglin/CD105	32.838	46.276	40.949	I15-I16	RAGE	3.294	18.046	15.363
D21-D22	Endostatin	10.421	4.927	18.920	**I17-I18**	**RBP4**	**25.513**	**6.256**	**23.195**
D23-D24	Fetuin A/AHSG	26.089	33.887	61.895	I19-I20	Reg3G	38.160	9.868	22.367
E1-E2	FGF acidic	13.748	30.504	23.084	I21-I22	Resistin	-41.256	52.610	22.900
E3-E4	FGF-21	-0.106	19.174	33.912	J1-J2	Reference Spots	0	0	0

E5-E6	Flt-3 Ligand	9.161	13.038	35.948	J3-J4	E-Selectin/CD62E	-3.206	11.958	22.972
E7-E8	Gas 6	7.481	4.587	25.967	**J5-J6**	**P-selectin/CD62P**	**32.712**	**18.010**	**43.120**
E9-E10	G-CSF	7.325	12.915	14.966	**J7-J8**	**Serpin E1/PAI-1**	**23.695**	**5.125**	**37.866**
E11-E12	GDF-15	16.209	1.502	23.689	J9-J10	Serpin F1/PEDF	-7.387	16.871	-26.145
E13-E14	GM-CSF	5.059	-20.070	48.559	**J11-J12**	**Thrombopoietin**	**22.567**	**22.917**	**9.826**
E15-E16	HGF	7.434	3.830	57.104	J13-J14	TIM-1/KIM-1/ HAVCR	-8.051	10.727	13.702
**E17-E18**	**ICAM-1/CD54**	**72.657**	**-0.128**	**82.491**	**J15-J16**	**TNF-alpha**	**5.704**	**20.574**	**30.554**
**E19-E20**	**IFN-gamma**	**-1.861**	**-19.765**	**28.068**	**J17-J18**	**VCAM-1/CD106**	**38.018**	**-2.701**	**22.062**
E21-E22	IGFBP-1	-9.399	72.233	329.654	**J19-J20**	**VEGF**	**34.211**	**20.947**	**44.648**
E23-E24	IGFBP-2	-15.007	-1.654	26.179	J21-J22	WISP-1/CCN4	14.164	10.383	17.146
F1-F2	IGFBP-3	2.971	12.213	40.932	J23-J24	Reference Spots	0	0	0
F3-F4	IGFBP-5	3.943	13.454	41.133					

Bold text: The factors with verification using enzyme-linked immunosorbent assay. ACT, Triterpenoids separated from *A. cinnamomea* mycelia; Sil, Silibinin.

Table 4s. ACT and Sil had no significant effect on the levels of inflammatory cytokines in liver of mice with acute alcohol injury.

	CTRL	Alcohol	Alcohol + Sil (mg/kg)	Alcohol + ACT (mg/kg)		
			63	5	15	45
TPO (pg/mgprot)	28.6±0.9	29.4±0.5	27.7±1.1	28.1±0.5	29.1±0.6	28.6±0.3
RBP4 (μg/mgprot)	8.9±0.3	9.6±0.3	9.1±0.3	9.2±0.2	9±0.2	8.8±0.4
IL-23 (pg/mgprot)	8.5±0.8	10.5±0.4	9.9±1.2	10±0.4	9.8±0.9	9.5±0.4
ICAM-1 (ng/mgprot)	78.6±7.2	68.9±2.1	77.7±8.1	79.4±2.3	73±3.5	73.7±3.9
NGAL (ng/mgprot)	3.5±0.4	3.5±0.4	3.2±0.3	3.3±0.1	3.3±0.3	2.9±0.1
VCAM-1(ng/mgprot)	46±2.7	52.9±1.3#	48.6±3.6	54.6±1.7	47.8±3.4	50.9±1.1

Data are expressed as the means ±S.D. (n=8) and were analyzed using a one-way analysis of variance (ANOVA) with parametric tests. # $P < 0.05$ *vs.* the control group; ACT: Triterpenoids separated from *A. cinnamomea* mycelia; Sil, Silibinin.

Supplementary figure legend

Figure 1s. The detail of mass spectrum detected by LC-MS/MS from ACT sample. Among of 25 types of triterpenoid compounds, fragment ions are not found in desoxylimonin, ganoderiol I, ganoderiol G and physalin D. Red arrow show fragment ion peak of compound. ACT, Triterpenoids separated from *A. cinnamomea* mycelia; LC-MS/MS, liquid chromatograph mass spectrometer/mass spectrometer 2. ① Ganoderal A; ② Ganoderol A; ③ Camelledionol; ④ Ganoderol B; ⑤ Camellenodiol; ⑥ Porrigenin A; ⑦ Tyromycic acid; ⑧ Ganoderal B; ⑨ Ganodermanondiol; ⑩ Lucidenic acid N; ⑪ Lucidenic acid M; ⑫ Glabrolide; ⑬ Desoxylimonin; ⑭ Rubinic acid; ⑮ Momoridcin; ⑯ Ganolucidic acid E; ⑰ Ganoderiol D; ⑱ Ganoderiol H; ⑲ Ganolucidic acid B; ⑳ Ganoderiol I; ㉑ Protobassic acid; ㉒ Ganoderiol G; ㉓ Phytolaccinic acid; ㉔ Tsugaric acid B; ㉕ Physalin D.

Figure 2s. ACT alone failed to influence the organ structures. Histopathological analysis in the (A) liver, (B) kidney, (C) spleen and (D) heart via H&E staining (scale bar: 100 μm; magnification: 400×). ACT, Triterpenoids separated from *A. cinnamomea* mycelia; Sil, Silibinin; H&E, hematoxylin and eosin.
