# Supplementary figures and images for: Triterpenoids Extracted From Antrodia cinnamomea Mycelia Attenuate Acute Alcohol-Induced Liver Injury in C57BL/6 Mice via Suppression Inflammatory Response
Source: Front Microbiol. 2020 Jul 3;11:1113. doi: 10.3389/fmicb.2020.01113 (PMC7350611; doi:10.3389/fmicb.2020.01113)

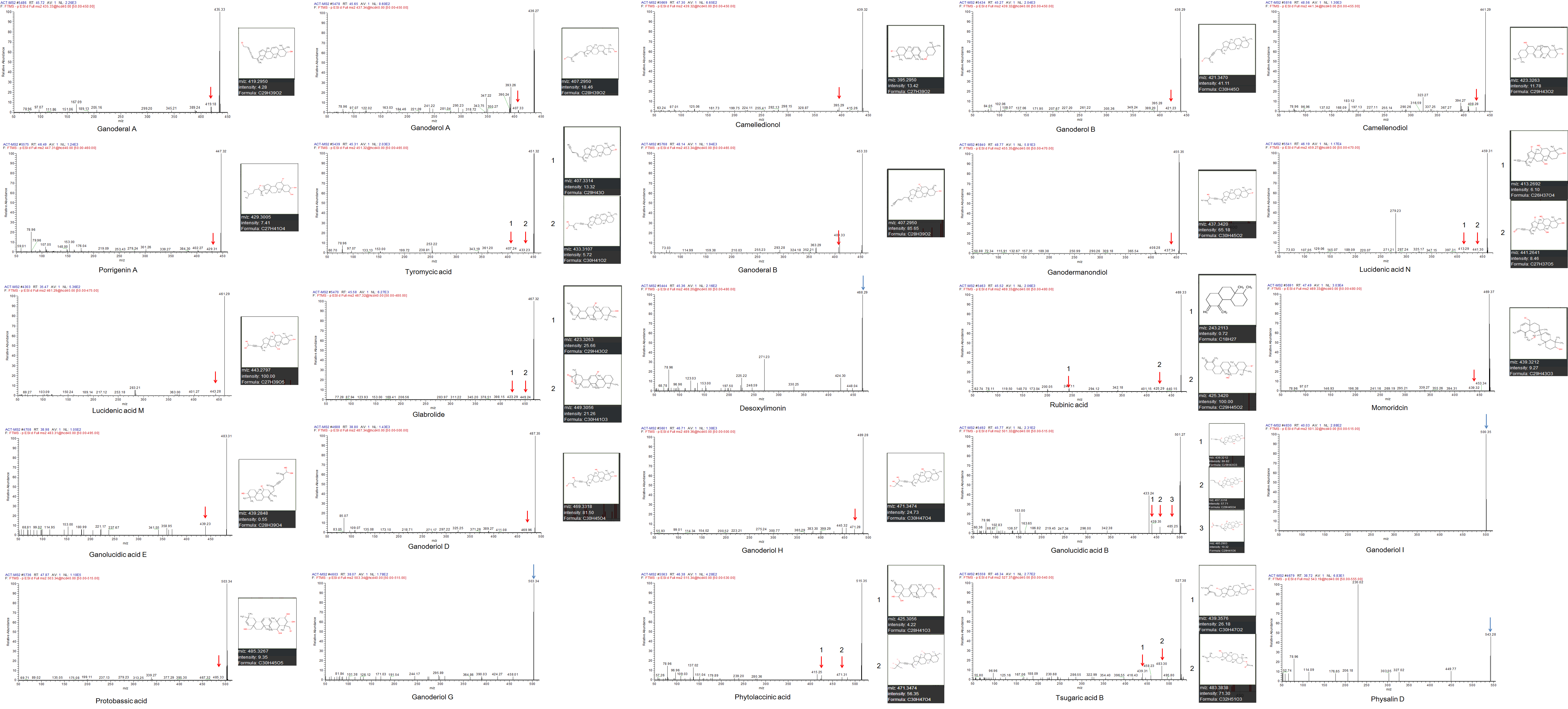

Supplement: Supplementary file 2 [file Image_1.jpg]

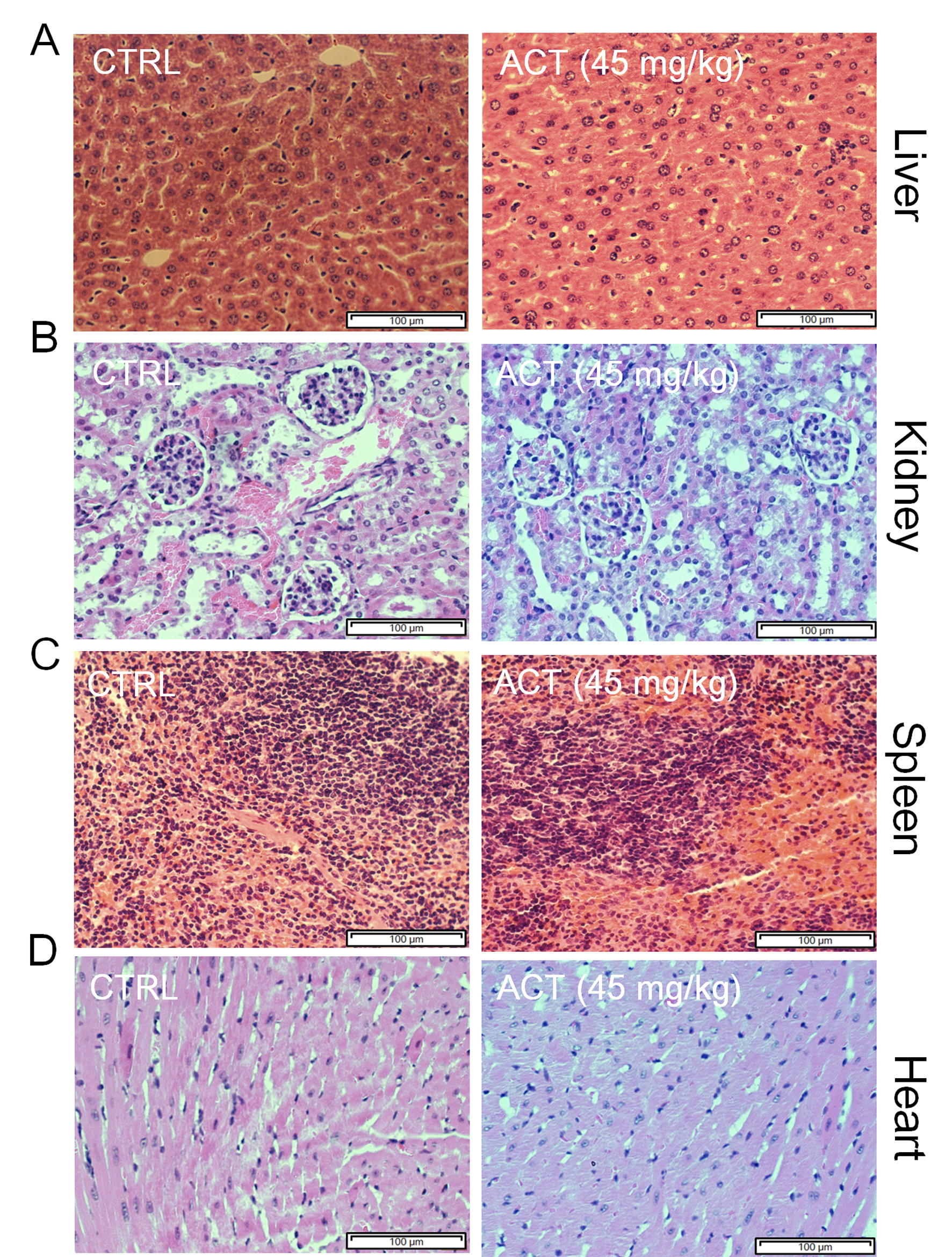

Supplement: Supplementary file 3 [file Image_2.TIF]
